# The Physician in Hawaii

**Published:** 1915-05

**Authors:** E. S. Goodhue

**Affiliations:** Holualoa, Hawaii


### The Physician in Hawaii.

BY E. S. GOODHUE, M. D., LL. D., HOLUALOA, HAWAII.

In complying with your request for a contribution, it has occurred to me that perhaps something describing the life and work of a country doctor in Hawaii would interest your readers more than a strictly scientific paper. At any rate, I am going to take the chances.

When I came here about twenty years ago there were fewer physicians in the field; each government doctor had his own district, which was large and fairly remunerative. He was paid a good salary, received his monthly check from the several sugar plantations by which he was employed, and made more or less from his private practice.

The government salaries have gradually been reduced, plantations do not pay much, and there may be several physicians dividing up the private practice. Since annexation, then, the Hawaiian medical positions are not sinecures.

Where there are large sugar plantations, or in districts which give the physician a good income from private work, the government salary has been cut down to practically nothing. Elsewhere it ranges from $100 to $150 a month with drugs.

Besides attending to all indigent patients of whatever nationality, the government medical man acts as registrar of marriages, births and deaths, is sanitary inspector and coroner. He must do post-mortems, testify in government cases, and do any other similar work he may be called upon to perform in emergencies.

My district, for instance, is some fifteen miles wide by three times that in length. One principal government road runs through the whole, and along it are small villages and scattered houses of whites—Hawaiians, Portuguese, Japanese, Chinese, Koreans, Filipinos, Porto Ricans and others.

Five well-conducted and housed schools are attended by about five hundred children of the aforementioned nationalities, while a few Japanese and Korean schools are maintained. It is my duty to visit these schools yearly, enforce vaccination, and see to general sanitary needs. Also, I address the children upon good citizenship, Abraham Lincoln and kindred subjects.

The principal calls for medicine and attendance are from the native Hawaiians. The most of them live along the beaches, or on trails which run up and down the mountain at rather regular intervals. Some occupy grass huts, but more live comfortably in modern houses of wood. Often you will come across the owners of a fine house squatted on the ground floor, on mats; preferring this to the wood floors above.

While Hawaii is generally salubrious and my district particularly heathful climatically, the usual diseases are to be found among the poor, except malaria, which has never occurred here. Colds, bronchitis, sometimes pneumonia, rheumatism, indigestion. We had a severe typhoid epidemic in 1909 with sporadic cases until last year. Asthma is common, chronic nephritis sometimes found, and an occasional appendicitis.

Cuts, bruises, infections, fractures, as elsewhere, of course. Measles, whooping cough, chickenpox and mumps appear now and then, but I think usually in a much milder form than on the mainland.

In the last ten years diphtheria has made its appearance, while scarlet fever has been reported from Honolulu. Dr. Manson says that the disease does not occur in the tropics, and I am inclined to believe he is right. I have not seen the disease here, and those who claim they have probably saw a mixed infection.

Follicular tonsilitis is quite common, and, among Japanese children, tetanus.

Infantile paralysis, of course, reached us with the usual rate of mortality and maiming.

Leprosy is confined almost altogether to the well equipped home for lepers on the Island of Molokai. It was my privilege to spend a week there a few months ago. The various hospitals and homes are in charge of the Sisters of Charity, whose kindly and efficient ministrations are very grateful to the inmates. These and others living outside to the number of several hundred seem very cheerful. Schools, churches, stores and places of amusement are provided. There is a brass band led by a blind leper who, by the way, composed music for one of my "songs," and had the band play it for me while I was there. I have sent some three hundred lepers to the settlement since my incumbency here.

At Kalawao, three miles distant from the settlement proper, is the United States Leprosy Investigation Station, and here at one of the homes resides the well known and interesting Joseph Dutton. Having given up his life to the service of the lepers, he has been the recipient of many honors from all parts of the world. Lately the G. A. R., of which he is a member, passed complimentary resolutions and sent him. I spent a very pleasant half hour with him, and shall long remember his cordial and courteous reception.

Medical calls here may come at any time, but, as a rule, my outside work is done in the forenoon, when it never rains. Office work afterwards and an occasional night call, but very seldom.

The physician is not called to a confinement case unless there be *pilikia,* or trouble. This is generally serious. Difficult presentations—placenta previa, retained placenta, eclampsia or hemorrhage. I attended a case of triplets the other day.

The whites seldom come to your office even for toothache, but call you ten, twenty or forty miles to their homes. Until lately our roads have been good, and I run my auto easily from one end of my district to the other. If the patient lives along a trail, I leave my auto at the road and generally find a horse or mule waiting to take me over the trail. The ride up or down is pleasant, through coffee trees, lantana and guava bushes. A large portion of my district is set out to coffee, and several hundred thousand dollars of the berry is sent abroad. Kona coffee is considered as good as any in the world.

Not far from my home is Kealakekua Bay, where Captain Cook was killed. His monument, erected in 1824 by Lord Byron, is there to mark the spot. It is rather the event, as no one knows the exact spot where the famous Englishman fell. His body was immediately spirited away by the natives and lain, probably, in one of the many caves hereabout. Tall cocoanut palms wave above the monument, and the bay glistens in eternal calm.

Ruins of old temples are found along the coast, burial caves, and piles of rock commemorating some event.

The white residents have comfortable and sometimes luxurious homes throughout the whole district, and came here from our own country, England, Germany, France, and other parts of the world. They are engaged for most part in buying and selling coffee, cattle raising and other occupations common to rural districts in the tropics.

Here are Episcopal, Catholics and Congregationalist Churches.

An efficient sanitary officer, D. S. Bowman, resides at Hilo, the metropolis of the island. He has the general superintendence of health matters of the island. The two districts of Kona have one hospital in common.

So far as private patients are concerned, in Hawaii their physician is never indispensable. He is a convenience rather than a tried and permanent friend and counselor of the family. "I hire him as I do my plumber," said one.

A new man is readily accepted when the old one goes. I sometimes think the change is rather gratifying to most patients; it has been the custom of the country for so long to change.

But the life grows on one here, and the conscientious physician learns to love the Hawaiians—to enjoy his contact with the various foreign races who come to regard him as a friend and helper. They at least believe in him. They trust his skill. They are always glad to see him and sorry to have him go. When I returned from a long absence once, a little Chinese woman rushed out to the road to greet me. "You come back—too muchee glad me." This sincere welcome came from the heart, due me for a little service I had rendered, but which had been paid for in money.

The Filipinos call the doctor "Papa," and seem to be grateful.

Often come gifts of fruit, vegetables, fresh meat, or a small pig. Last week from Hawaiians some fine woven mats, or it may be a hat made from the leaves of the pandanus.

But, as with other work in which the heart lies, the best reward is the satisfaction of doing. Just doing the very best one can, with thoroughness and sympathy, fretting never as to what gratiture may or may not follow.

Such rewards, the kind, health-giving climate, and a sense of home in this most delicious of lands, have kept me here away from tempting and remunerative offers in the cities of your mainland.

---

JUST LIKE A MULE.—"Oh, doctor, I have sent for you, certainly; still, I must confess that I have not the slightest faith in modern medical science."

"Well," said the doctor, "that doesn't matter in the least. You see, a mule has no faith in the veterinary surgeon, and yet he cures him all the same."—*Houston Chronicle.*

---

NEEDED A REST.—Doctor (after an examination): What is your business?

Patient: Me? Oh, I am a gentleman!

Doctor: I see! Well, you'll have to cut it out for six months. Go some place where you can get it completely off your mind. Say, Washington.—*Houston Chronicle.*

